# A High-Fat Diet Activates the BAs-FXR Axis and Triggers Cancer-Associated Fibroblast Properties in the Colon

**DOI:** 10.1016/j.jcmgh.2021.12.015

**Published:** 2021-12-29

**Authors:** Tae-Young Kim, Seungil Kim, Yeji Kim, Yong-Soo Lee, Sohyeon Lee, Su-Hyun Lee, Mi-Na Kweon

**Affiliations:** 1 Mucosal Immunology Laboratory, Department of Convergence Medicine, University of Ulsan College of Medicine/Asan Medical Center, Seoul; 2 Digestive Diseases Research Center, University of Ulsan College of Medicine, Seoul, Republic of Korea

**Keywords:** Cancer Stem Cells, Gut Microbiome, Mesenchymal Stromal Cells, Wnt2b, ANOVA, analysis of variance, BA, bile acid, BSH, bile salt hydrolase, CA, cholic acid, CAF, cancer-associated fibroblast, CDCA, chenodeoxycholic acid, CD44v, CD44 variant isoforms, CMP, cecal microbial products, CRC, colorectal cancer, CSC, cancer stem cell, DCA, deoxycholic acids, DMEM, Dulbecco modified Eagle medium, ECAR, extracellular acidification rate, ECM, extracellular matrix, FFA, free fatty acids, GSEA, gene set enrichment analysis, HFD, high-fat diet, ISC, intestinal stem cell, MSC, mesenchymal stromal cell, NCD, normal chow diet, OCR, oxygen consumption rate, PD, purified diet, qPCR, quantitative polymerase chain reaction, RNA-seq, RNA sequencing, RT, room temperature, SD, standard deviation

## Abstract

**Background & Aims:**

Dietary signals are known to modulate stemness and tumorigenicity of intestinal progenitors; however, the impact of a high-fat diet (HFD) on the intestinal stem cell (ISC) niche and its association with colorectal cancer remains unclear. Thus, we aimed to investigate how a HFD affects the ISC niche and its regulatory factors.

**Methods:**

Mice were fed a purified diet (PD) or HFD for 2 months. The expression levels of ISC-related markers, ISC-supportive signals, and Wnt2b were assessed with real-time quantitative polymerase chain reaction, in situ hybridization, and immunofluorescence staining. RNA sequencing and metabolic function were analyzed in mesenchymal stromal cells (MSCs) from PD- and HFD-fed mice. Fecal microbiota were analyzed by 16s rRNA sequencing. Bile salt hydrolase activity and bile acid (BA) levels were measured.

**Results:**

We found that expression of CD44 and Wnt signal-related genes was higher in the colonic crypts of HFD-fed mice than in those fed a PD. Within the ISC niche, MSCs were expanded and secreted predominant levels of Wnt2b in the colon of HFD-fed mice. Of note, increased energy metabolism and cancer-associated fibroblast (CAF)-like properties were found in the colonic MSCs of HFD-fed mice. Moreover, colonic MSCs from HFD-fed mice promoted the growth of tumorigenic properties and accelerated the expression of cancer stem cell (CSC)-related markers in colon organoids. In particular, production of primary and secondary BAs was increased through the expansion of bile salt hydrolase-encoding bacteria in HFD-fed mice. Most importantly, BAs-FXR interaction stimulated Wnt2b production in colonic CAF-like MSCs.

**Conclusions:**

HFD-induced colonic CAF-like MSCs play an indispensable role in balancing the properties of CSCs through activation of the BAs-FXR axis.

## Graphical abstract

**Figure:**
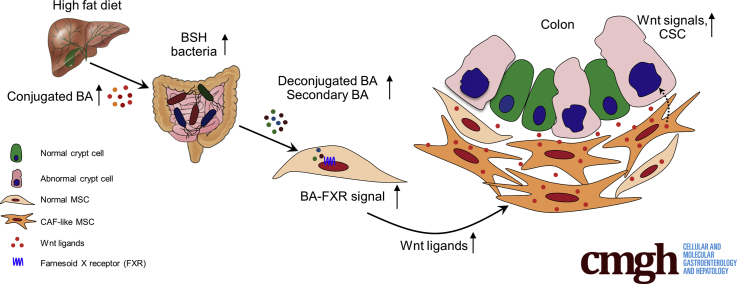

**Summary**

We observed the expansion of BSH-producing bacteria by a HFD increased deconjugated and secondary BAs in the colon. Deconjugated and secondary BAs stimulated Wnt2b production in colonic MSCs by activating FXR, thereby resulting in hyperproliferation of colonic crypts.

Diet influences a large proportion of stem cell maintenance and function in mammals, and biological changes in these stem cells may be associated with cancer incidence.^1^ Previous research has demonstrated that a high-fat diet (HFD) amplified the self-renewal of intestinal stem cells (ISCs) and progenitor cells and triggered their potential tumor-initiating properties.^2^ Similarly, cancer stem cells (CSCs) have been suggested as a driving force of tumor progression, long-term maintenance, and metastasis.^3^ Although CSCs have been detected and their function determined in various cancers, their response to dietary signals remains unclear.

The function of ISCs is sensitively regulated through cell-autonomous and non-autonomous mechanisms in response to various dietary signals.^4^, ^5^, ^6^ For example, the ketogenic diet increases stemness by controlling β-hydroxybutyrate production in ISCs.^4^ Dietary L-glutamate also enhances the proliferation of ISCs through the increase of cytosolic Ca^2+^ concentration.^7^ However, through a cell non-autonomous mechanism, calorie restriction enhances the function of ISCs by affecting neighboring Paneth cells.^5^ Although research indicates that dietary signals influence the ISC niche, their modulation of the niche function in the colon remains undetermined.

Mesenchymal stromal cells (MSCs), components of the ISC niche, are located within the subepithelium close to ISCs in the intestine. MSCs are known to provide signaling molecules such as Wnt ligands and R-spondins for ISC maintenance.^8^^,^^9^ Unlike the small intestine, the colon lacks Paneth cells, so colonic MSCs are the primary source of Wnts (eg, Wnt2b, Wnt4, and Wnt5a).^10^^,^^11^ Although MSCs support the maintenance of ISCs, they have duplicity in that they contribute to cancer development as components of the tumor microenvironment.^12^ Activated MSCs in the tumor microenvironment promote tumor development and progression through secretion of cytokines, growth factors, and extracellular matrix (ECM) as cancer-associated fibroblasts (CAFs).^13^, ^14^, ^15^ Despite the elucidation of the intrinsic effects of HFD on ISCs, the influence of extrinsic factors such as ISCs on HFD-induced intestinal tumorigenesis remains unclear.

Various dietary factors contribute to the formation of gut microbiota, which is closely related to host homeostasis. We previously demonstrated that gut microbiota-derived metabolites could modulate the function of ISCs and their niche.^16^^,^^17^ In addition, gut microbiota manipulate the metabolism of primary conjugated bile acids (BAs), which in turn generate secondary BAs.^18^ A HFD stimulated BA secretion into the intestine^19^ and expanded gut bacteria with enzymatic activity involved in BA metabolism, thereby increasing toxic secondary BAs.^20^^,^^21^ Recent studies have reported that activation of BA-responsive receptors such as FXR and TGR5 regulate ISC maintenance and epithelial regeneration.^22^^,^^23^ However, despite the established role of gut microbiota-derived BAs in ISC function and maintenance, the underlying mechanism of BAs on the regulation of the ISC niche remains undetermined.

In this study, we investigated the effect of a HFD on the ISC niche and its relation to colorectal cancer (CRC) risk. We observed that colonic MSCs from HFD-fed mice promoted crypt regeneration through Wnt2b production and expressed CAF-like properties. Importantly, the BAs-FXR axis accelerated the CAF-like properties of colonic MSCs. Thus, we suggest that a HFD stimulates the pro-tumorigenic function of MSCs by altering BA metabolism through the gut microbiota.

## Results

### HFD Leads to Pathologic Conditions and Tumorigenesis in the Colon

**Figure 1:**
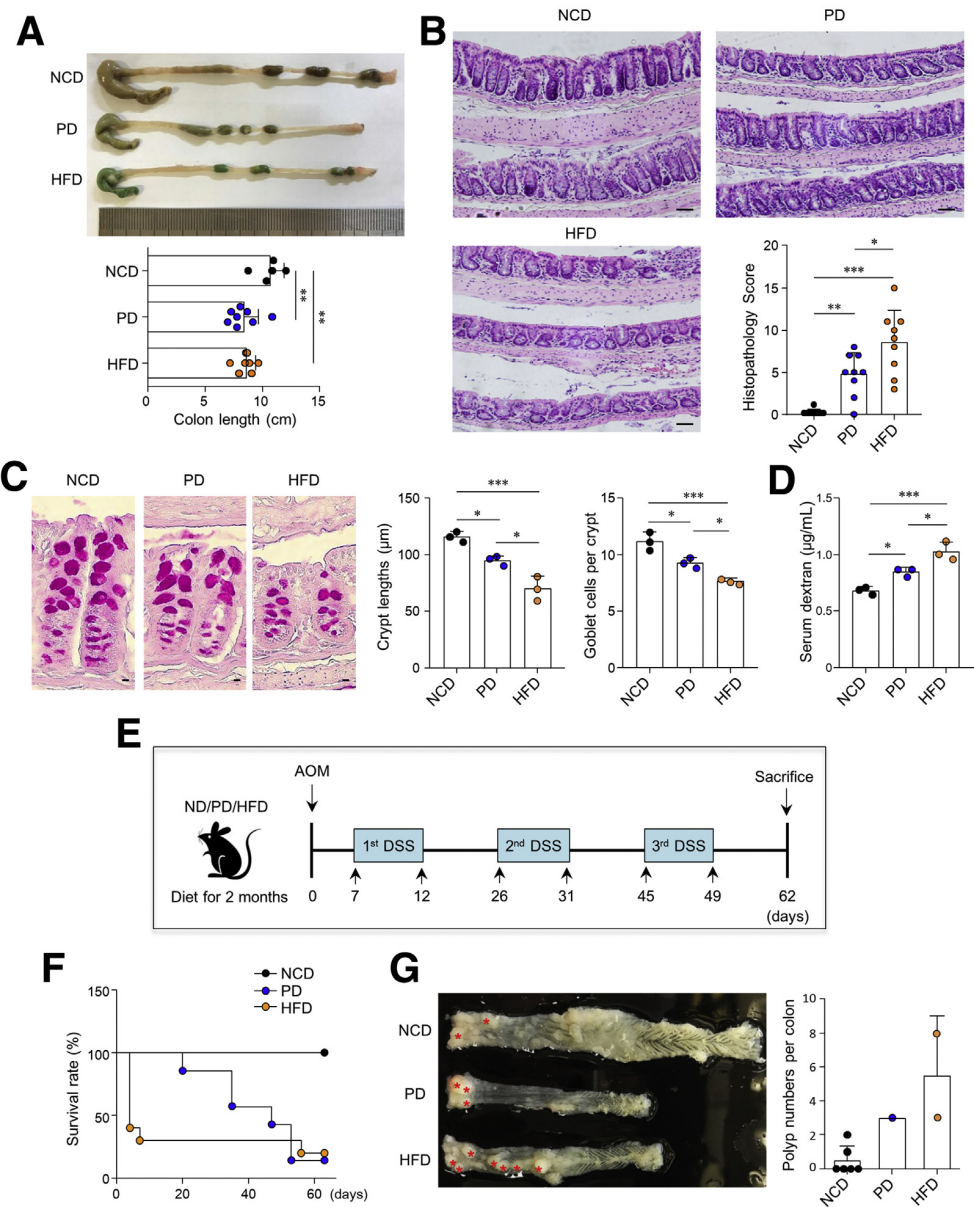
**HFD resulted in pathologic conditions in the colon.***(A*) Representative colon image and measurement of colon length from NCD-, PD-, and HFD-fed mice for 2 months. (*B*) Representative hematoxylin-eosin staining image and histopathology score from colon tissues. Scale bar = 50 μm. **(***C*) Representative periodic acid-Schiff (PAS) staining image, crypt lengths, and goblet cell number per crypt score from colon tissues. Crypt lengths and goblet cell number were measured at ≥35 crypts. Scale bar = 5 μm. **(***D*) Measurement of FITC in serum after FITC-dextran administration to PD- and HFD-fed mice. (*E*) Scheme of inflammation-induced colon tumorigenesis model made by AOM/DSS treatment after diet for 2 months. (*F*) Survival rate of PD- and HFD-fed mice after AOM/DSS treatment. (*G*) Representative image of colon and polyp numbers per colon. *Red asterisks* indicate polyps. Data are presented as mean ± standard deviation (SD); comparisons were made by two-tailed *t* test. ∗*P* < .05, ∗∗*P* < .01, ∗∗∗*P* < .001. Data were repeated 2 times in independent experiments. DSS, dextran sulfate sodium; FITC, fluorescein isothiocyanate.

To address the impact of HFD on colon physiology, we first fed mice a normal chow diet (NCD), purified diet (PD), or HFD for 2 months. Previous research has clarified that the choice of a refined vs a chow diet significantly affects gut microbiota composition and host physiology.^24^ We therefore selected PD as a control for the HFD. Both the PD and HFD were formulated with refined ingredients containing the same amount of fiber. Unexpectedly, shorter colon lengths were found in mice fed the PD and HFD compared with mice fed the NCD (Figure 1*A*). We further addressed pathologic changes of the colon by HFD feeding in a steady-state condition. Histopathology score of the colon was significantly higher in the HFD-fed mice than those in the PD- or NCD-fed mice (Figure 1*B*). In addition, shorter crypt lengths and reduced numbers of goblet cells were found in the HFD-fed mice (Figure 1*C*). Gut permeability measured by serum dextran levels was higher in the HFD-fed mice than in the PD- or NCD-fed mice (Figure 1*D*). Of note, significant alteration of colon pathology was also detected in the PD-fed mice compared with those in the NCD-fed mice (Figure 1). To confirm whether HFD accelerates tumorigenesis as reported previously,^2^^,^^25^ we administered Azoxymethane (AOM) and dextran sulfate sodium to each diet-fed mice to induce inflammation-driven CRC (Figure 1*E*). Almost all HFD-fed mice died within a few days of treatment with AOM (10 mg/kg body weight), whereas all NCD-fed mice survived (Figure 1*F*). In addition, PD-fed mice had a lower survival rate than NCD-fed mice (Figure 1*F*). The surviving HFD-fed mice (n = 2/10) possessed more polyps in the colon than mice fed the NCD or PD (Figure 1*G*). Similar to the steady-state, the colon length of mice fed the PD and HFD was shorter than that of mice fed the NCD (Figure 1*G*). Overall, we confirmed that the HFD induced pathologic changes and promoted inflammation-induced tumorigenesis in the colon. Feeding of the PD caused mild pathologic changes in the colon; therefore, we adopted the PD as a control for the subsequent experiments.

### HFD Amplifies CD44 Expression and Wnt Signaling in Colonic Crypts

**Figure 2:**
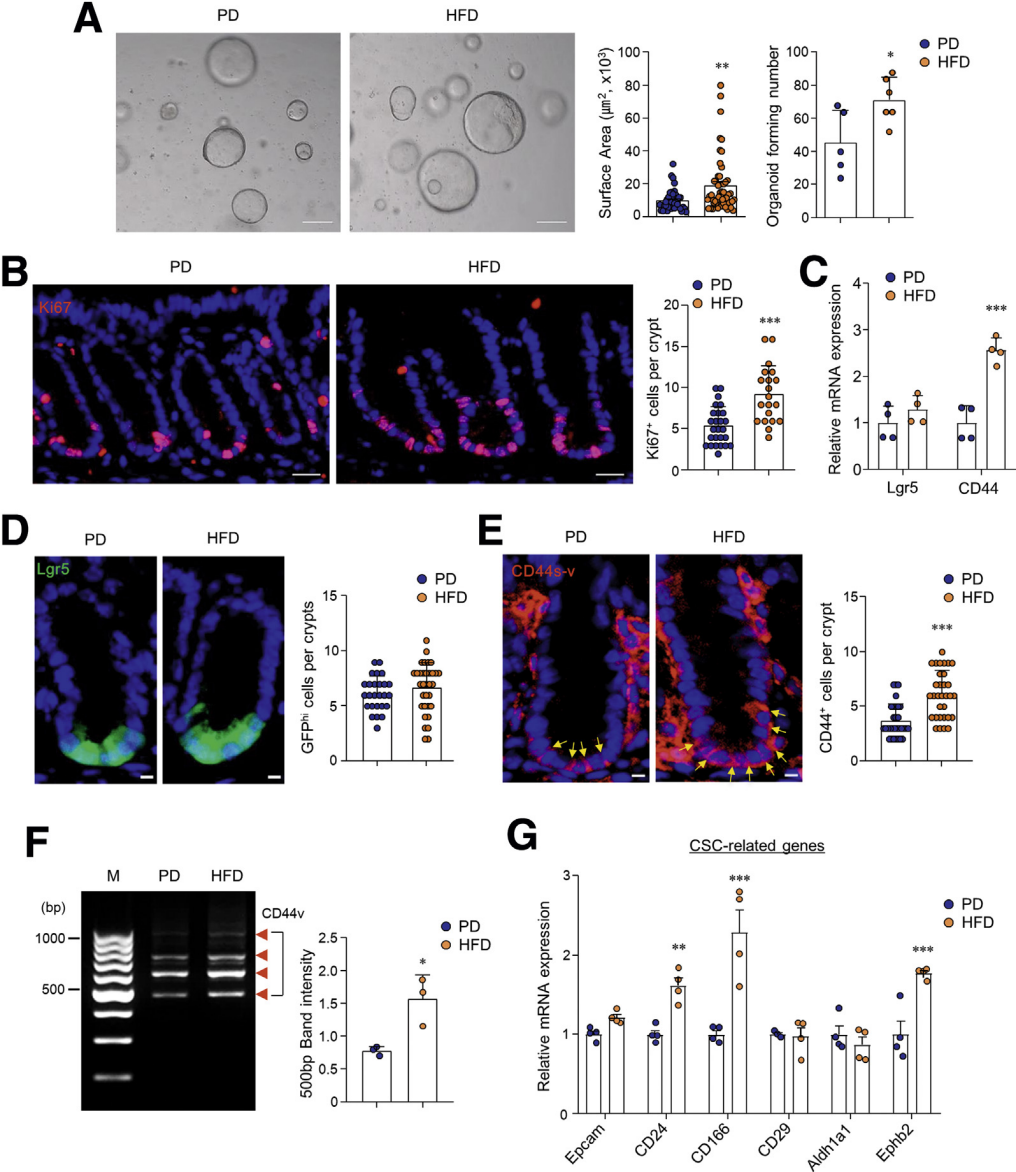
**HFD induced abnormal stemness and CSC.** (*A*) Colon organoids were cultured from PD- and HFD-fed mice. Scale bar = 200 μm. Surface area and the forming number of organoids were measured. Surface area was measured at ≥70 organoids. (*B*) Immunofluorescence image of nuclei (*blue*) and Ki67 (*red*) in colon and quantification of Ki67 positive cells from the confocal image. Scale bar = 20 μm. (*C*) Relative mRNA expression levels of genes related to ISCs were measured by real-time PCR in the colonic crypts. (*D*) Immunofluorescence image of nuclei (*blue*) and Lgr5 (*green*) in the colon of PD- and HFD-fed Lgr5-EGFP mice and quantification of GFP^high^ cells in the crypts. Scale bar = 5 μm. (*E*) Immunofluorescence image of nuclei (*blue*) and CD44s-v (*red*) in the colon and quantification of CD44 positive cells per crypts from the confocal image. CD44 antibody recognizes an epitope on both CD44 standard forms (CD44s) and CD44 isoforms (CD44v). *Yellow arrows* indicate CD44 positive cells. Scale bar = 20 μm. (*F*) Real-time PCR analysis of total mRNA from colonic crypts of PD- and HFD-fed mice for primers targeted to exon 5 and 16 of the *CD44* and *β-actin*. M = DNA size marker. (*G*) Relative mRNA expression levels of genes related to CSC markers were measured in the colonic crypts. Data are presented as mean ± SD; comparisons were made using the two-tailed *t* test or two-way ANOVA multiple test. ∗*P* < .05, ∗∗*P* < .01, ∗∗∗*P* < 0.001. Data were repeated twice in independent experiments.

**Figure 3:**
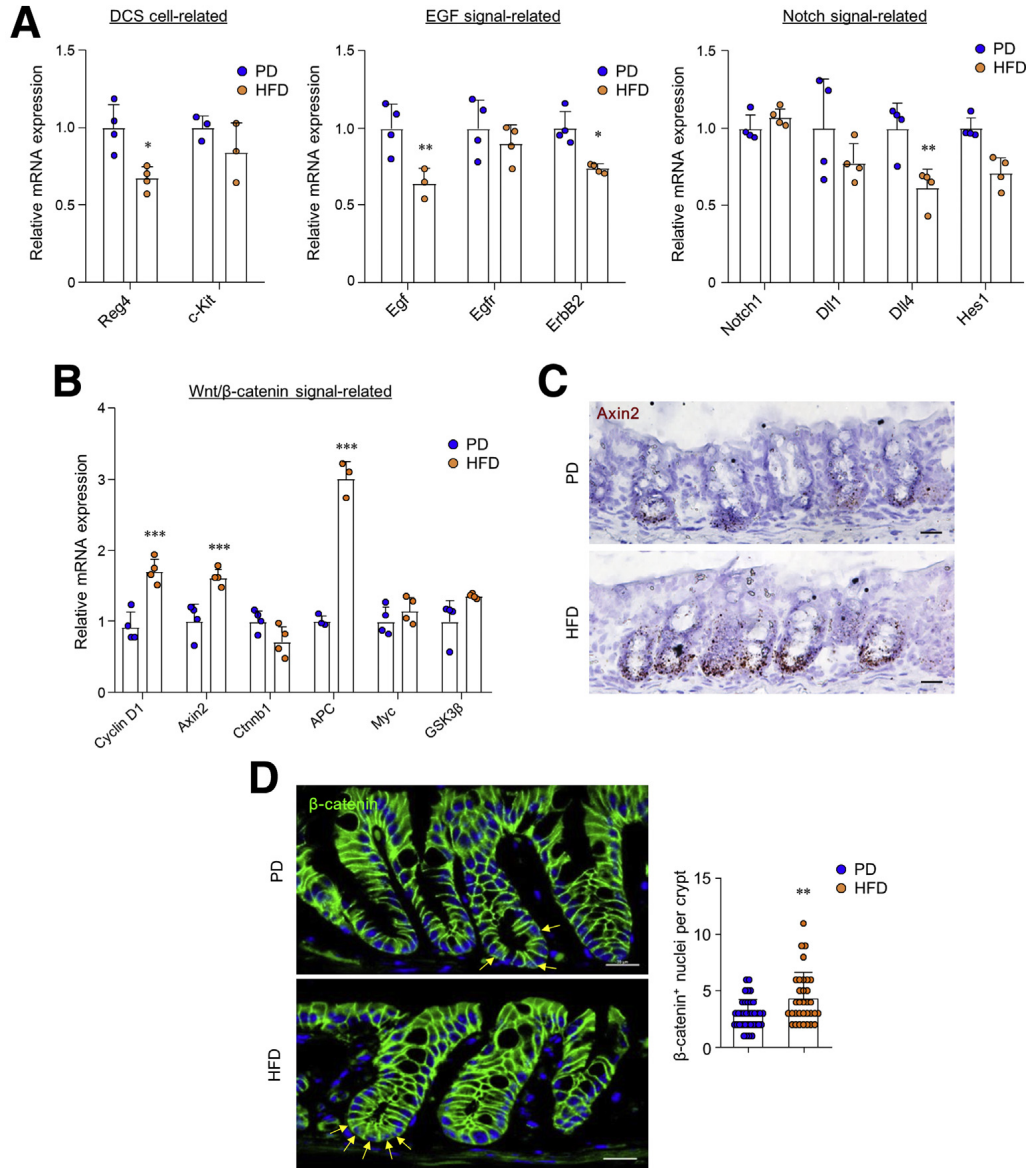
**HFD amplified Wnt/β-catenin signaling in colonic crypts.** (*A*) Relative mRNA expression levels of genes related to deep crypt secretory cells, EGF signals, and Notch signals were measured in the colonic crypts. (*B*) Relative mRNA expression levels of genes related to Wnt signaling pathways were measured by real-time PCR in the colonic crypts. (*C*) Representative images show *Axin2* in situ hybridization in the colon. Scale bar = 20 μm. (*D*) Immunofluorescence image of nuclei (*blue*) and β-catenin (*green*) in colon and quantification of nuclear β-catenin localization from the confocal image. *Yellow arrows* indicate nuclear translocation of β-catenin. Scale bar = 20 μm. Data are presented as mean ± SD; comparisons were made using two-tailed *t* test or two-way ANOVA multiple test. ∗*P* < .05, ∗∗*P* < .01, ∗∗∗*P* < .001. Data were repeated twice in independent experiments.

Next, we investigated the effects of a HFD on the regeneration of colonic crypts. Using an ex vivo three-dimensional culture system, we assessed the ability of isolated colonic crypts to form organoids. In line with previous data,^2^ mice fed a HFD produced significantly enhanced organoids in size and formation compared with those fed the PD (Figure 2*A*). In addition, Ki67-positive proliferating cells in the ISC and progenitor-dense crypt region of the colon increased significantly in HFD-fed mice compared with PD-fed mice (Figure 2*B*). On the basis of the activity of crypt regeneration, we examined the expression levels of ISC markers, leucine-rich-repeat-containing G-protein-coupled receptor 5 (Lgr5) and CD44, in the colonic crypts of PD- and HFD-fed mice. Although expression levels of Lgr5 did not differ, CD44 expression was significantly higher in the colonic stem cell regions of HFD-fed mice than those in PD-fed mice (Figure 2*C* and *D*). Interestingly, previous studies have identified various isoforms of the CD44 family, which are formed by mRNA splicing and are known as CSC markers for CRC.^26^^,^^27^ We found that the numbers of CD44-positive cells and CD44 variant isoforms (CD44v) were augmented in the colonic crypts of the HFD-fed mice than in the PD-fed mice (Figure 2*E* and *F*). In addition, representative CSC markers (ie, CD24, CD166, and Ephb2) increased significantly in the colonic crypts of HFD-fed mice compared with those in PD-fed mice (Figure 2*G*). However, mRNA expression associated with deep crypt secretory cell markers, epidermal growth factor, and Notch signaling, which supports ISC maintenance, showed no difference or decreased (Figure 3*A*). Among Wnt/β-catenin–related genes, *Cyclin D1, Axin2*, and *APC* were significantly higher in HFD-fed mice than in PD-fed mice (Figure 3*B*). Hyperexpression levels of *Axin2* in the colons of HFD-fed mice were confirmed with in situ hybridization (Figure 3*C*). We next assessed higher nuclear β-catenin accumulation in the colonic crypts of HFD-fed mice compared with those of PD-fed mice (Figure 3*D*). These results support the notion that a HFD increases stemness and expression of CSC markers through overexpression of Wnt/β-catenin signaling in the colonic crypt.

### HFD Stimulates Wnt2b Production and Cancer-Associated Properties in Colonic MSCs

**Figure 4:**
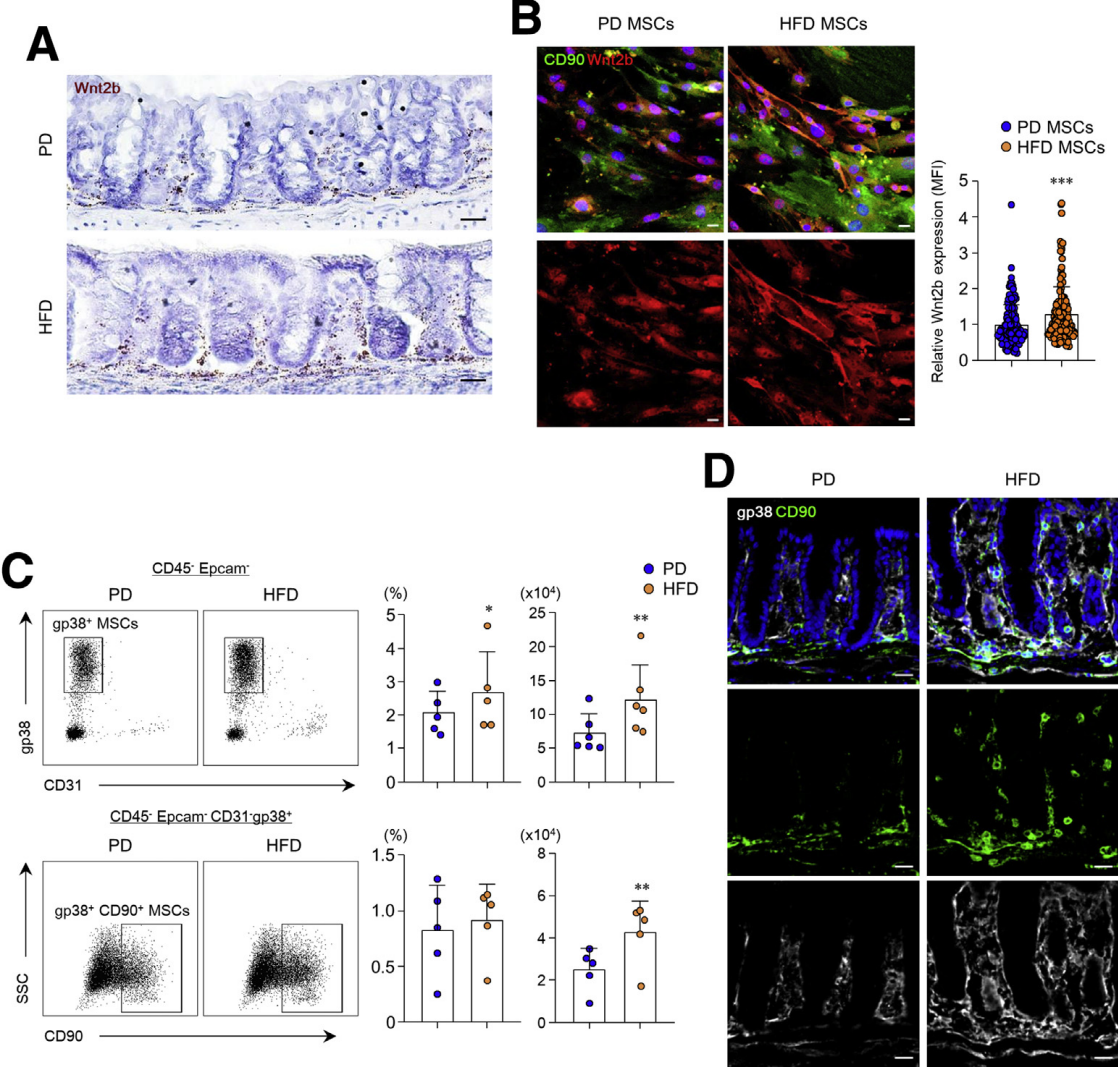
**Wnt2b production and expansion of colonic MSCs were promoted by HFD.** (*A*) Representative images show Wnt2b in situ hybridization in colon of mice fed PD or HFD. Scale bar = 20 μm. (*B*) Immunofluorescence image of nuclei (*blue*), CD90 (*green*), and Wnt2b (*red*) in MSCs and quantification of Wnt2b expression were measured through relative Wnt2b mean fluorescence intensity (MFI). Scale bar = 20 μm. (*C*) Representative fluorescence-activated cell sorter plots, percentages, and absolute numbers of gp38^+^ MSCs and gp38^+^CD90^+^ MSCs in total recovery cells. (*D*) Immunofluorescence image of nuclei (*blue*), CD90 (*green*), and gp38 (*white*) in colon of mice fed PD or HFD. Scale bar = 20 μm. Data are presented as mean ± SD; comparisons were made with two-tailed *t* test. ∗*P* < .05, ∗∗*P* < .01, ∗∗∗*P* < .001. Data were repeated twice in independent experiments.

**Figure 5:**
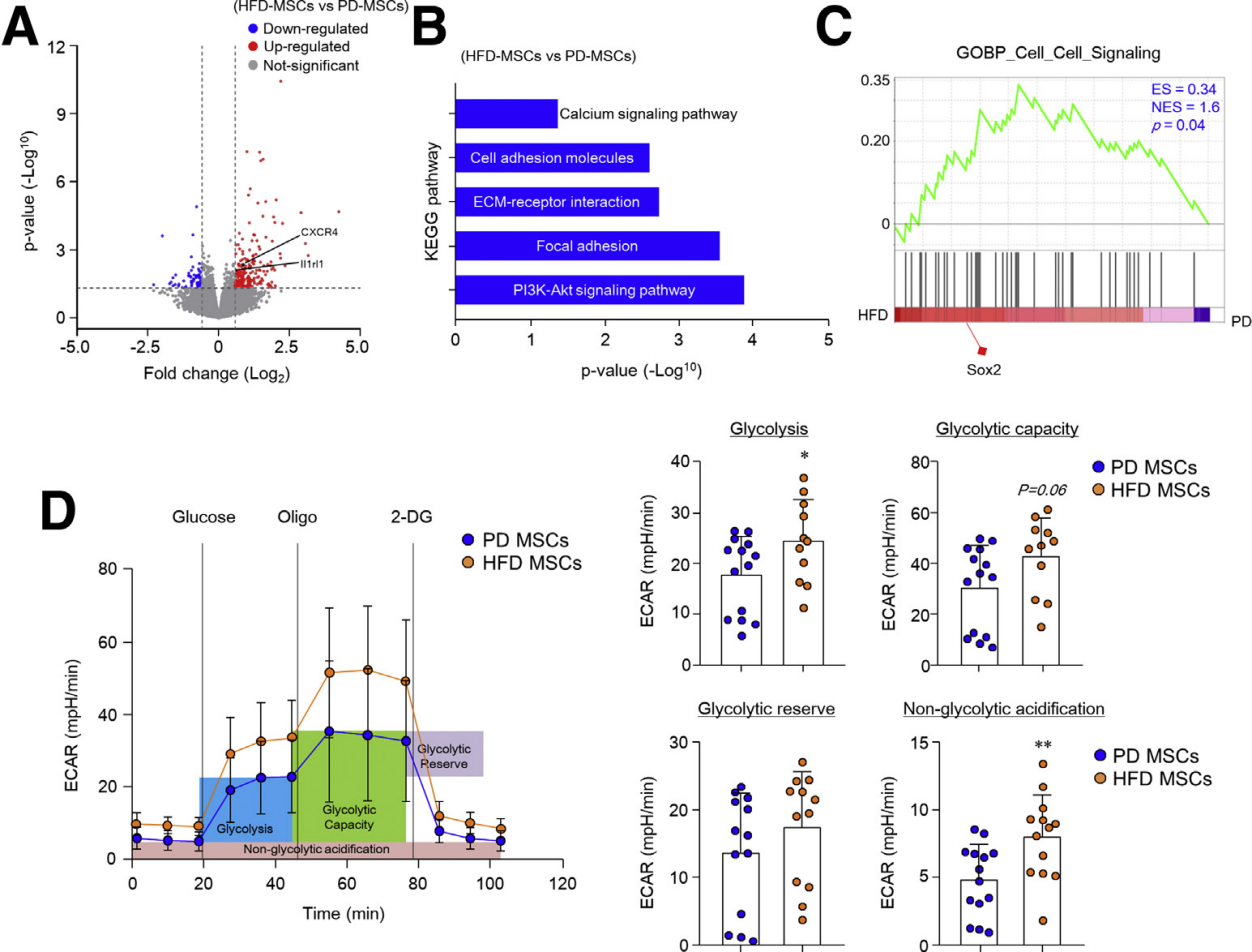
**HFD-derived colonic MSCs have CAF-like properties.** (*A–C*) RNA-seq analysis of colonic MSCs cultured from PD- and HFD-fed mice. (*A*) Volcano plots of all genes based on their log2 fold-change and *P* values. Differentially expressed genes (fold-change ≥ 1.5 ; *P* < .05) were sorted. (*B*) KEGG pathway analysis through gProfiler (fold-change ≥ 1.5 ; *P* < .05). (*C*) GSEA in colonic MSCs (fold-change ≥ 1.5). Enrichment score (ES), normalized enrichment score (NES), and *P* values are stated in the panel, and representative genes are indicated under the panel. (*D*) ECAR of colonic MSCs was measured in response to injection of glucose, oligomycin (Oligo), and 2-deoxy-glucose (2-DG) under basal conditions. Parameters of glycolysis stress were measured from the ECAR of colonic MSCs. Data are presented as mean ± SD; comparisons were made with two-tailed *t* test. ∗*P* < .05, ∗∗*P* < .01, ∗∗∗*P* < .001. Data were repeated twice in independent experiments.

We hypothesized that colonic MSCs could potentially activate Wnt/β-catenin signaling under conditions of a HFD. Higher expression of Wnt2b was determined in the subepithelium and submucosa of the colon, where MSCs are usually localized, from mice fed with a HFD than from mice eating a PD (Figure 4*A*). On the other hand, there was a slight increase in Wnt5a in the colon subepithelium of mice fed a HFD, but not other Wnt family members (ie, Wnt2, Wnt3, Wnt4, Wnt6, Wnt9b) (data not shown). Wnt2b expression was primarily observed in the presence of ISC and progenitor cells in the crypt bottom; therefore, Wnt2b rather than Wnt5a may play an essential role in the maintenance and development of colonic stem cells. Recent studies have suggested that gp38^+^CD90^+^ MSCs produce Wnt2b, a critical growth factor for ISC maintenance.^28^ Therefore, we assessed the Wnt2b expression levels in colonic CD90^+^ MSCs. Of interest, Wnt2b production was significantly higher in colonic MSCs isolated from mice fed a HFD than PD-fed mice (Figure 4*B*). Fluorescence-activated cell sorter and immunohistochemistry analysis revealed higher numbers of gp38^+^CD90^+^ MSCs in the subepithelial region of mice fed a HFD than a PD (Figure 4*C* and *D*). To further investigate the alteration of colonic MSCs by HFD, we performed RNA-sequencing (RNA-seq) experiments. Among the 290 genes that differ significantly between the 2 groups, 226 were up-regulated in colonic MSCs from HFD-fed mice compared with PD-fed mice (Figure 5*A*). Notably, predominant levels of CXCR4 and IL1RL1 expression, which are known to activate MSCs to CAFs,^29^^,^^30^ were identified in colonic MSCs by HFD feeding. KEGG pathway analysis of HFD-derived MSCs showed up-regulation of the PI3K-Akt signaling pathway, focal adhesion molecules, ECM-receptor interaction, cell adhesion molecules, and the calcium signaling pathway (Figure 5*B*). In gene set enrichment analysis (GSEA), we found increased expression of Sox2 in colonic MSCs from HFD-fed mice (Figure 5*C*), which reportedly promotes tumorigenesis.^31^ We then measured the oxygen consumption rate (OCR) (an indicator of oxidative phosphorylation) and extracellular acidification rate (ECAR) (an indicator of glycolysis) to fully assess the effects of a HFD on the energy metabolism of colonic MSCs. Although there was no significant difference in OCR level (data not shown), ECAR level after treatment with glucose was enhanced in HFD-derived MSCs compared with PD-derived MSCs (Figure 5*D*). In addition, as parameters of glycolysis stress, glycolysis, glycolytic capacity, and non-glycolytic acidification increased in colonic MSCs after HFD feeding (Figure 5*D*). Considering these results, we concluded that HFD stimulates colonic MSC expansion, Wnt2b production, and cancer-associated properties.

### HFD-Derived MSCs Induce CSC Characteristics in Colon Organoids

**Figure 6:**
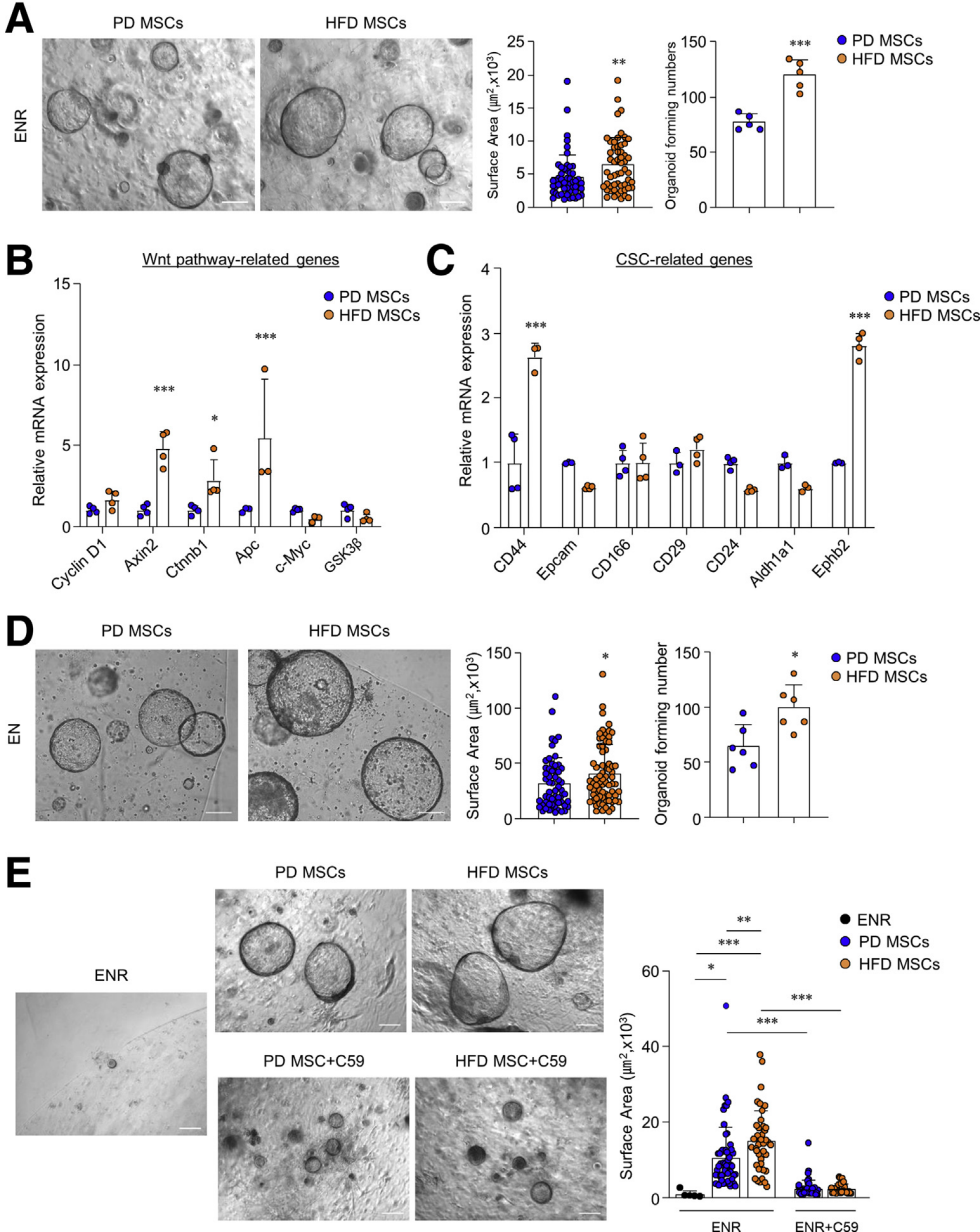
**HFD-derived colonic MSCs stimulate proliferation and CSCs gene expression in colon organoids.** (*A*) Colon organoids from naive C57BL/6N mice were co-cultured with MSCs from PD- and HFD-fed mice in ENR medium. Scale bar = 200 μm. Surface area and forming number of organoids were measured. Surface area was measured at ≥50 organoids. (*B*) Relative mRNA expression levels of genes related to Wnt signal pathways were measured in the naive colon organoids co-cultured with colonic MSCs. (*C*) Relative mRNA expression levels of CSC markers were measured in the naive colon organoids co-cultured with colonic MSCs. (*D*) Colon organoids from *Apc*^*Min/+*^ mice were co-cultured with MSCs in EN medium. Scale bar = 200 μm. Surface area and forming number of organoids were measured. Surface area was measured at ≥50 organoids. (*E*) Colon organoids from naive C57BL/6N mice were co-cultured with MSCs in ENR medium or ENR containing C59 for 9 days. C59 was treated only for the final 2 days. Scale bar = 200 μm. Surface area of organoids was measured. Surface area was measured at ≥25 organoids. Data are presented as mean ± SD; comparisons were made by two-tailed *t* test or two-way ANOVA multiple test. ∗*P* < .05, ∗∗*P* < .01, ∗∗∗*P* < .001. Data were repeated twice in independent experiments.

We next investigated whether HFD-derived MSCs promote colon organoid growth and formation. In the presence of ENR medium, HFD-derived MSCs increased both size and formation of organoids compared with PD-derived MSCs (Figure 6*A*). We next investigated whether colonic MSCs from HFD-fed mice directly stimulated Wnt/β-catenin signaling and CSC markers expression. The Wnt signal target genes were analyzed from colon organoids co-cultured with MSCs obtained from each group of mice. *Axin2*, *Ctnnb1*, and *Apc* expression had increased significantly in organoids co-cultured with MSCs from HFD-fed mice compared with those from PD-fed mice (Figure 6*B*). Of note, higher mRNA levels of CD44, CD166, and Ephb2 were observed in organoids co-cultured with HFD-derived MSCs than in PD-derived organoids (Figure 6*C*). To examine whether HFD-derived colonic MSCs promote tumorigenesis, we co-cultured the colon organoids derived from *Apc*^*Min*/+^ mice with colonic MSCs from each group of mice. Co-culture with HFD-derived MSCs promoted the size and formation of colon organoids derived from *Apc*^*Min*/+^ mice in the presence of ENR medium (Figure 6*D*). To clarify the effect of Wnt proteins secreted by MSCs on tumorigenic organogenesis, Wnt inhibitors (ie, C59) were added to the ENR medium. As anticipated, the growth of organoids co-cultured with MSCs of HFD-fed mice decreased significantly, similar to as much as organoids co-cultured with MSCs of PD-fed mice (Figure 6*E*). These results indicate that HFD promotes proliferation of ISCs and CSCs through stimulation of Wnt2b secretion by colonic MSCs.

### Metabolites From Altered Gut Microbiota by HFD Caused Hyper Wnt2b Secretion From MSCs

**Figure 7:**
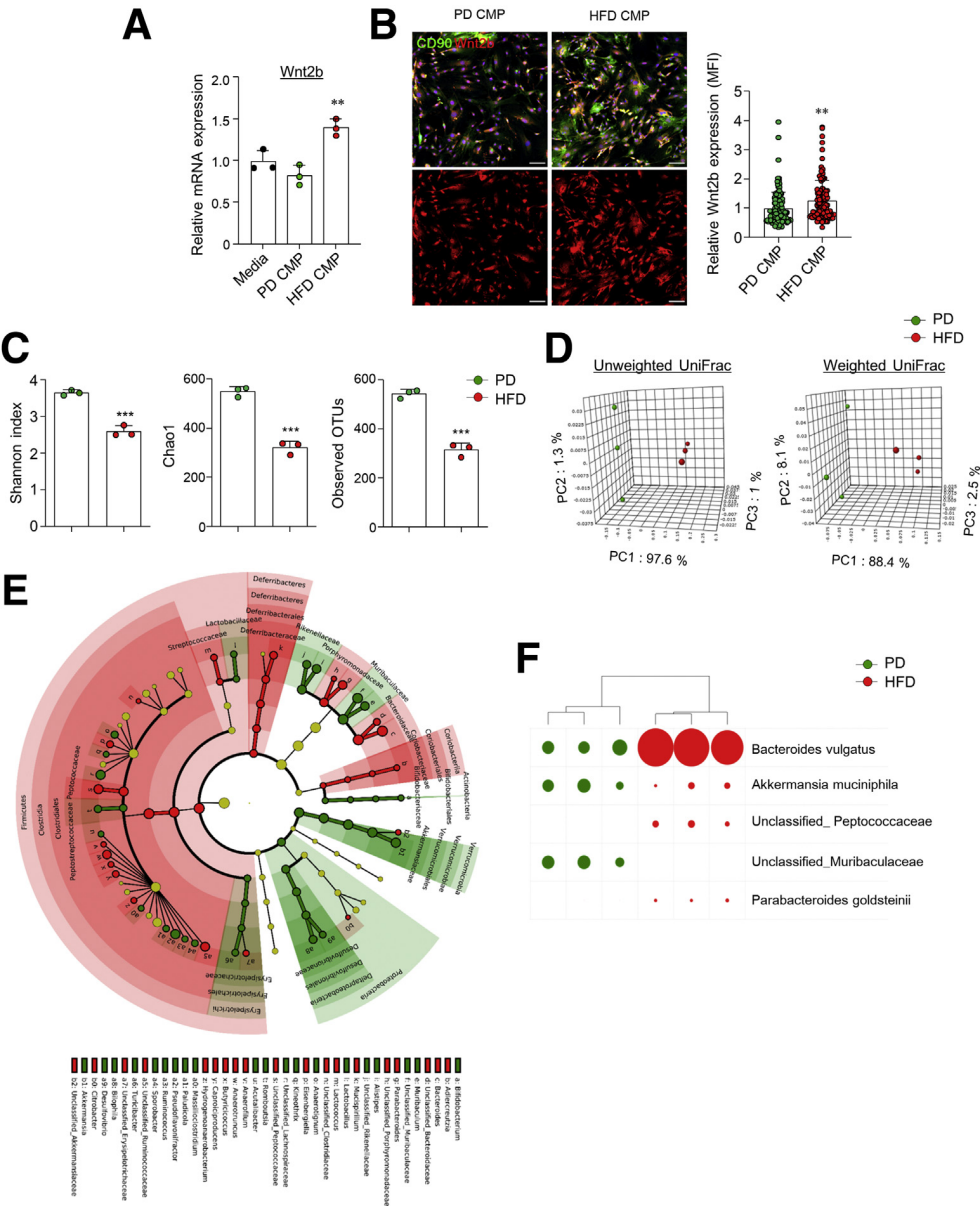
**HFD led to a change in gut microbiota composition and community.** (*A*) Relative mRNA expression levels of Wn2b were measured in the naive in vitro colonic MSCs treated with CMP from PD- and HFD-fed mice. (*B*) Immunofluorescence image of nuclei (*blue*), CD90 (*green*), and Wnt2b (*red*) in the in vitro colonic MSCs treated with CMP and relative Wnt2b mean fluorescence intensity (MFI) was calculated on the basis of the control group. Scale bar = 100 μm. (*C*–*F*) Pyrosequencing analysis for gut microbiota after a HFD was determined from feces of PD- and HFD-fed mice. Shannon index, Chao1, and operational taxonomic units (OTUs) of fecal microbiota were determined (*C*). Principal component analysis (PCA) of unweighted UniFrac and weighted UniFrac distances was analyzed for clustering fecal microbial communities (*D*). Taxonomic cladogram from LEfSe and dot size means proportional to taxon abundance (*E*). Comparison of bacterial ratio at the species level. Size of circle equates proportion in entire (*F*). Full size of circle = 45.41%. Bacteria with minimum ratio >2% only were compared. Data are presented as mean ± SD; comparisons were made by two-tailed *t* test or two-way ANOVA multiple test. ∗∗*P* < .01. Data were repeated twice in independent experiments.

Alteration of microbial metabolites by HFD feeding is associated with intestinal tumorigenesis.^25^ Therefore, we next determined the regulation of gut microbial metabolites on MSC dysfunction in HFD-fed mice. Colonic MSCs were obtained from naive C57BL/6N mice and treated with cecal microbial products (CMP) from PD- and HFD-fed mice. Interestingly, CMP from HFD-fed mice stimulated more Wnt2b expression by colonic MSCs than that from PD-fed mice (Figure 7*A* and *B*). We next analyzed the profiles of gut microbiota in the feces of PD- and HFD-fed mice. The results of the Shannon index, Chao1, and operational taxonomic units indicated that the diversity of resident gut microbiota decreased in the feces of HFD-fed mice compared with those of PD-fed mice (Figure 7*C*). Principal component analysis of unweighted and weighted UniFrac revealed clear separation of gut microbiota composition between PD- and HFD-fed mice (Figure 7*D*). In LEFSe taxonomic cladogram, the gut microbiota of HFD-fed mice were dominated by the Firmicutes and Deferribacteres phyla, with lower proportions of Proteobacteria and Verrucomicrobia phyla (Figure 7*E*). Interestingly, *Bacteroides vulgatus* increased significantly in HFD-fed mice compared with PD-fed mice at the species level (Figure 7*F*). Overall, these results imply that alteration of microbial metabolites by HFD feeding may drive the activation of colonic MSCs.

### HFD-Derived Gut Microbiota Increased the BA Metabolites

**Figure 8:**
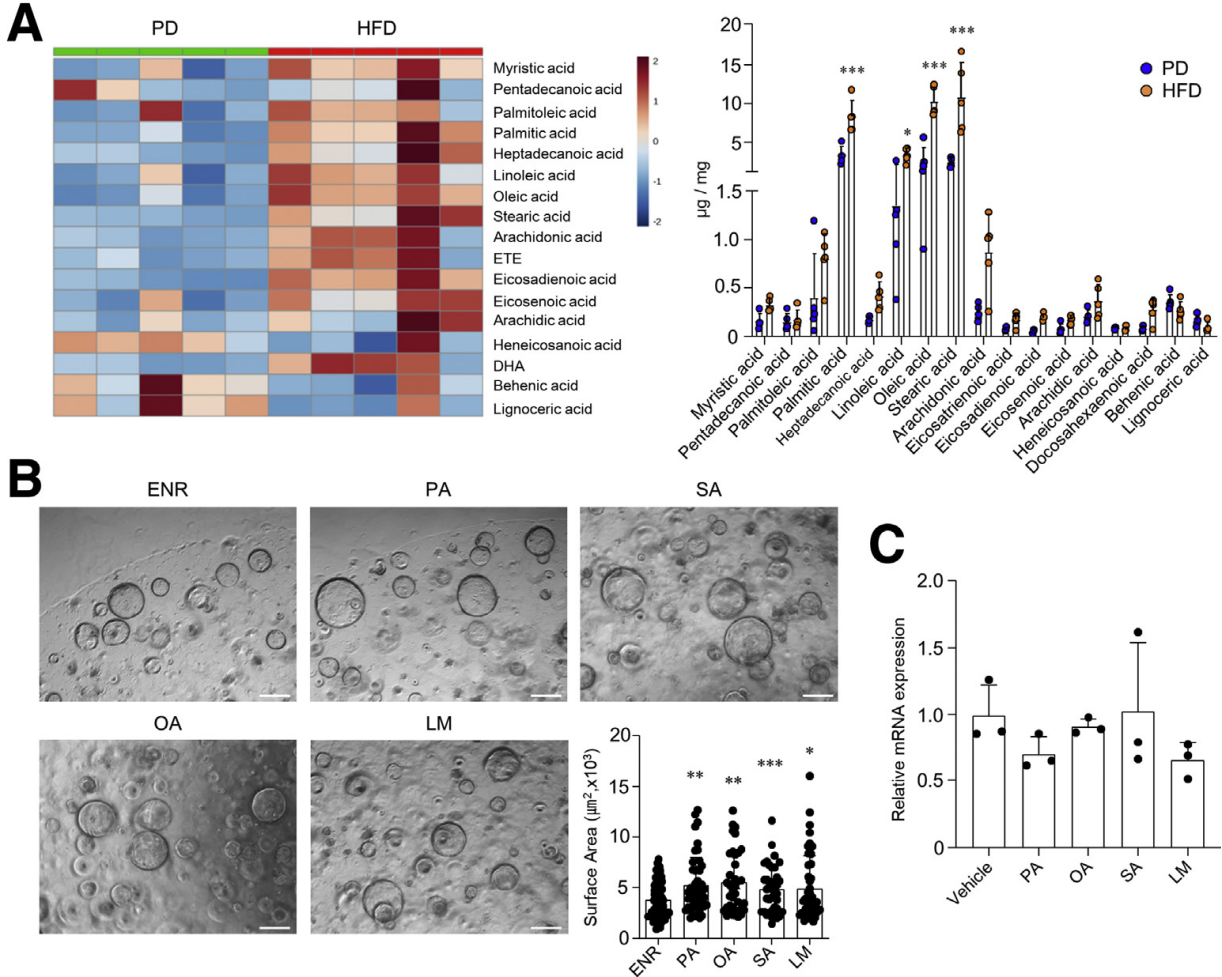
**FFA did not promote Wnt2b production from colonic MSCs.** (*A*) FFA levels were analyzed in CMP of PD- and HFD-fed C57BL/6N mice for 5 months. (*B*) Colon organoids of naive C57BL/6N mice were cultured with palmitic acid (PA; 30 μmol/L), stearic acid (SA; 30 μmol/L), oleic acid (OA; 30 μmol/L), or lipid mixture (LM; 1%). Scale bar = 200 μm. Surface area was measured at ≥40 organoids. (*C*) Relative mRNA expression levels of Wnt2b were measured in colonic MSCs treated with FFAs (30 μmol/L PA, 30 μmol/L OA, 30 μmol/L SA, 1% LM). Data are presented as mean ± SD; comparisons were made with two-tailed *t* test or two-way ANOVA Bonferroni multiple test. ∗*P* < .05, ∗∗*P* < .01, ∗∗∗*P* < .001. Data were repeated twice in independent experiments.

**Figure 9:**
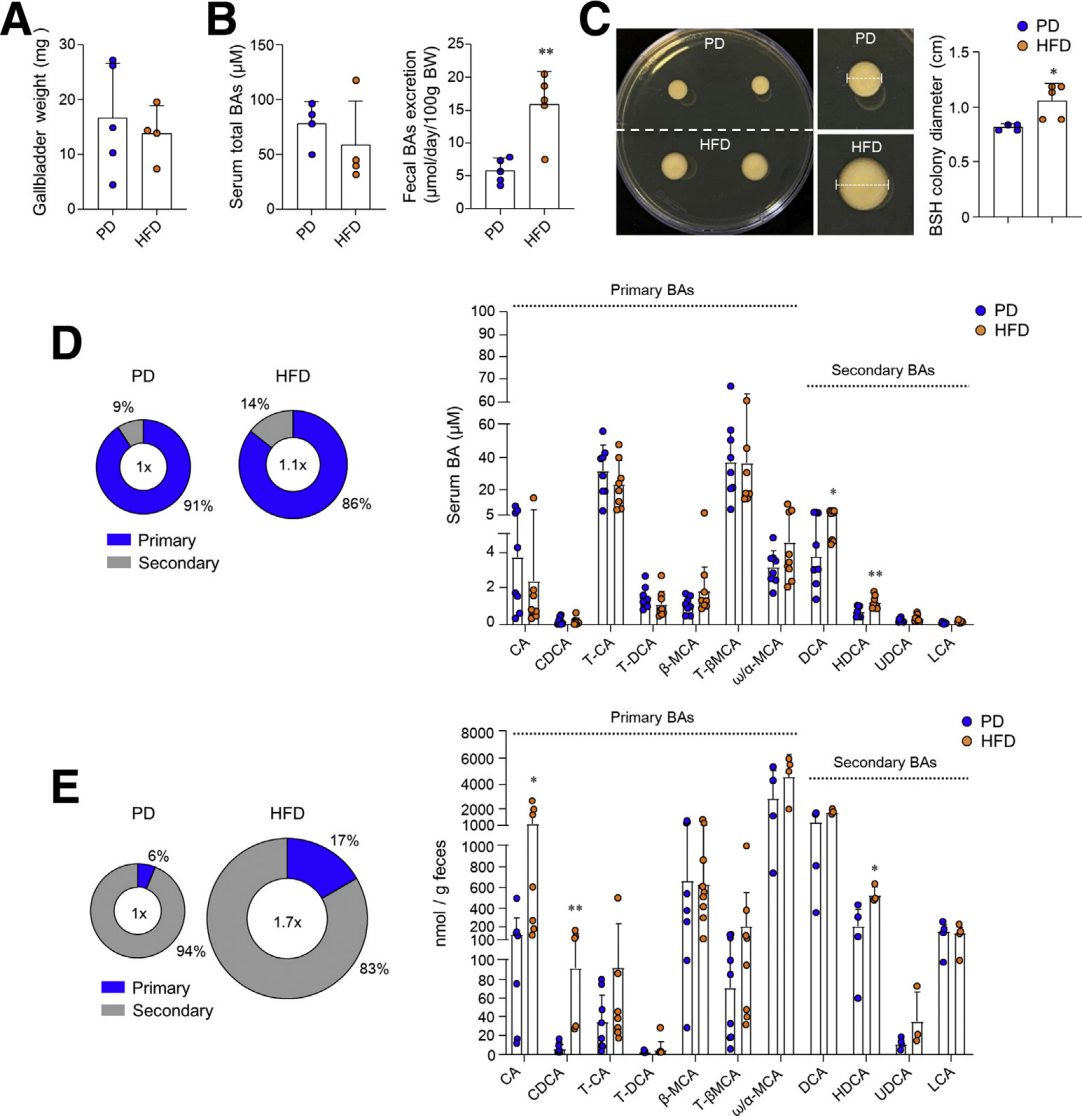
**HFD alters composition of BAs through gut microbiota.** (*A*) Weights of gallbladders in mice fed PD or HFD for 2 months. (*B*) Total BA levels were measured in the portal vein and feces. Fecal BA excretion was calculated from fecal total BA levels. (*C*) BSH activity assay in feces. (*D*) Serum BA composition and individual BA levels were measured. (*E*) Fecal BA composition and individual BA levels were measured. Data are presented as mean ± SD; comparisons were made with two-tailed *t* test or two-way ANOVA multiple test. ∗*P* < .05, ∗∗*P* < .01. Data were repeated twice in independent experiments.

To identify the gut microbiota-derived metabolites that play a critical role in MSCs activation, we first analyzed free fatty acids (FFAs) in the cecal content. As expected, overall the proportion of FFAs was significantly higher in the cecal content of HFD-fed mice compared with that from PD-fed mice (Figure 8*A*). As reported previously,^2^ each FFA (ie, palmitic acid, oleic acid, stearic acid, and the mixture combination) enhanced colon stemness (Figure 8*B*). However, each FFA could not stimulate Wnt2b secretion from MSCs (Figure 8*C*). We next considered BAs as another candidate of gut microbiota-derived metabolites. We observed no difference in gallbladder weight between the PD- and HFD-fed mice (Figure 9*A*). Thus, we investigated potential differences in the amount of BAs excreted from the gallbladder. Although there was no difference in serum BA levels, fecal BA excretion was significantly higher in HFD-fed mice than in PD-fed mice (Figure 9*B*). We next cultured feces in MRS agar containing taurodeoxycholic acids to assess the activity of the bile salt hydrolase (BSH) responsible for BA deconjugation. As shown in Figure 9*C*, the tauro-precipitated colony was significantly larger in HFD-fed mice than in PD-fed mice. Therefore, we analyzed BA composition in serum and feces to determine whether a HFD affects BA metabolism by altering the gut microbiota. The proportion of secondary BAs (ie, deoxycholic acid [DCA] and hyodeoxycholic acid) in the serum was higher in HFD-fed mice than in PD-fed mice (Figure 9*D*). However, the proportion of primary BAs (ie, cholic acid [CA] and chenodeoxycholic acid [CDCA]) and secondary BAs (ie, hyodeoxycholic acid) in the feces was greater in the HFD-fed mice compared with the PD-fed mice (Figure 9*E*). These results demonstrated that a HFD expanded BSH-producing bacteria and subsequently increased the production of deconjugated and secondary BAs.

### BAs-FXR Axis Stimulates Wnt2b Production in Colonic MSCs

**Figure 10:**
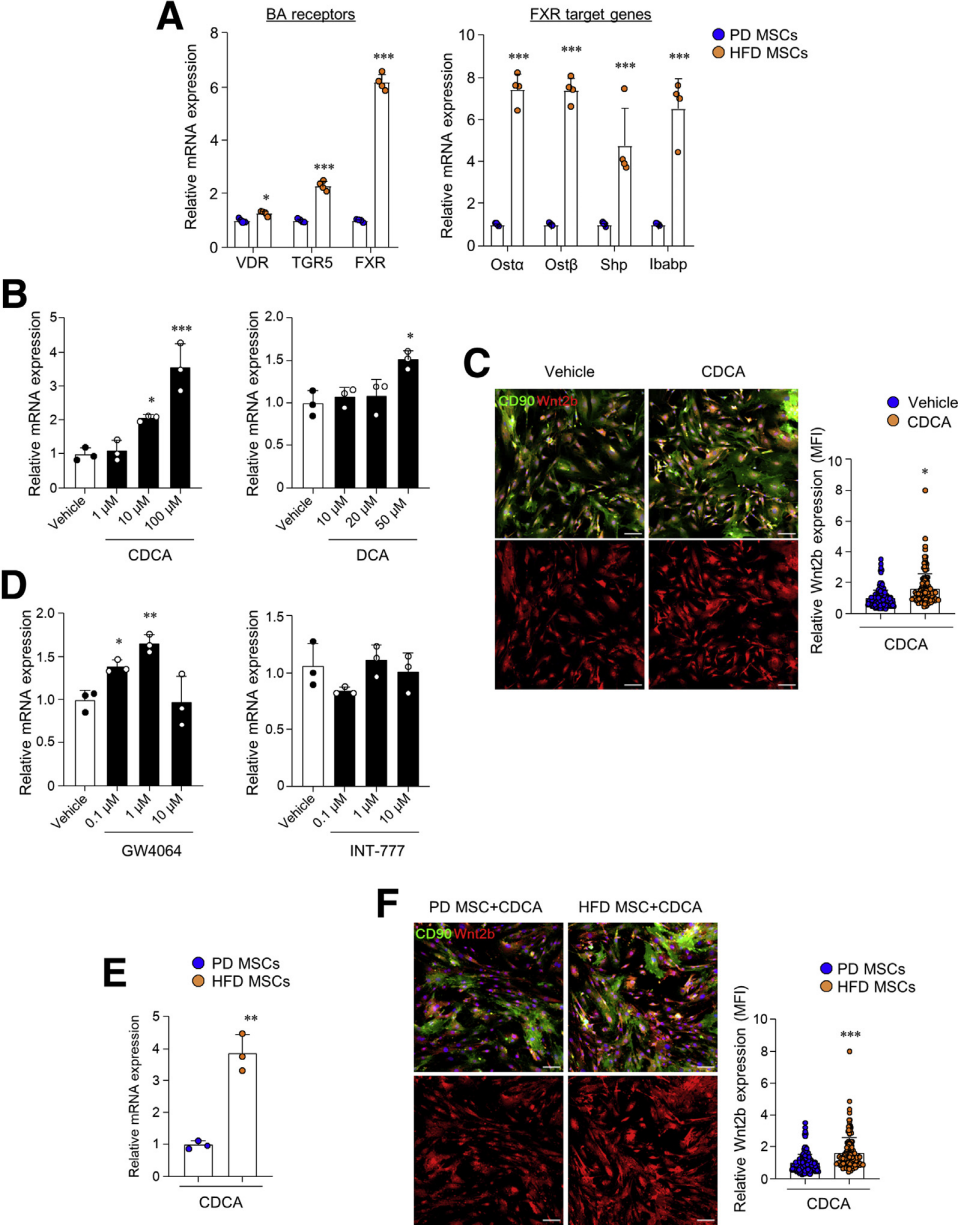
**Activated FXR signal pathway triggers Wnt2b production in colonic MSCs.** (*A*) Relative mRNA expression levels of genes related to BA receptors and FXR signaling target genes were measured in colonic MSCs obtained from PD- and HFD-fed mice. (*B*) Relative mRNA expression levels of Wn2b were measured in colonic MSCs treated with CDCA or DCA. (*C*) Immunofluorescence image of nuclei (*blue*), CD90 (*green*), and Wnt2b (*red*) in colonic MSCs treated with CDCA, and relative Wnt2b mean fluorescent intensity (MFI) was calculated on the basis of the control group. Scale bar = 100 μm. (*D*) Relative mRNA expression levels of Wn2b were measured in naive in vitro colonic MSCs treated with GW4064 or INT-777. (*E*) Relative mRNA expression levels of Wn2b were measured in colonic MSCs treated with CDCA. (*F*) Immunofluorescence image of nuclei (*blue*), CD90 (*green*), and Wnt2b (*red*) in colonic MSCs treated with CDCA, and relative Wnt2b MFI was calculated on the basis of the control group. Scale bar = 100 μm. Data are presented as mean ± SD; comparisons were made using two-tailed *t* test, one-way ANOVA, or two-way ANOVA multiple test. ∗*P* < .05, ∗∗*P* < .01, ∗∗∗*P* < .001. Data were repeated twice in independent experiments.

On the basis of the increased levels of BAs in HFD-fed mice, we investigated the expression levels of BA receptors in colonic MSCs. The proportion of all types of BA receptors (ie, VDR, TGR5, and FXR) was significantly higher in colonic MSCs obtained from HFD-fed mice than those obtained from PD-fed mice (Figure 10*A*). In addition, expression of FXR-target genes (ie, OSTα, OSTβ, SHP, and IBABP) increased significantly by HFD feeding (Figure 10*A*). We then co-cultured colonic MSCs with CDCA or DCA, natural ligands with a high affinity for BA receptors, and analyzed the expression of Wnt2b to determine the effects of BAs on Wnt2b secretion from colonic MSCs. CDCA treatment greatly increased the expression of Wnt2b from colonic MSCs isolated from naive mice in a dose-dependent manner (Figure 10*B)*. We also found similar effects of DCA, another natural ligand for the BA receptor (Figure 10*B)*. In addition, increased protein levels of Wnt2b expression by CDCA were also observed (Figure 10*C*). Because CDCA and DCA are known to have an affinity for both FXR and TGR5,^32^ we adopted specific agonists for FXR (ie, GW4064) or TGR5 (ie, INT-777) to identify the receptor signaling that primarily mediates increased Wnt2b expression in vitro*.* GW4064 significantly increased Wnt2b mRNA expression, whereas INT-777 did not affect Wnt2b, indicating FXR rather than TGR5 plays a critical role in the Wnt2b production by HFD feeding (Figure 10*D*). Interestingly, HFD-derived MSCs promoted Wnt2b production to a greater extent than PD-derived MSCs in the presence of CDCA (Figure 10*E* and *F*). Overall, activation of BAs-FXR signaling stimulated Wnt2b production in colonic MSCs.

## Discussion

This study demonstrates the dysregulation of colonic stem cells and MSCs in mice fed a HFD through in vivo mouse model and ex vivo organoids studies. HFD feeding resulted in dysregulation of colonic MSCs, including increased cell numbers, overexpression of Wnt2b, and activation of CAF-like properties. Consistently, HFD-derived colonic MSCs triggered CSC properties and Wnt-related signals. Most importantly, we observed increased BA receptor expression on colonic MSCs and determined that BAs further stimulate colonic MSC-induced Wnt2b production through the activation of FXR signaling. Overall, our results imply that colonic MSCs play an indispensable role in altering CSCs in HFD-induced tumorigenesis through the BAs-FXR axis.

Earlier studies demonstrated that dietary factors could regulate tumorigenicity of ISCs or intestinal progenitors.^2^^,^^33^ The hierarchical CSCs model has been proposed to explain the development of tumorigenicity.^34^ As the origin of malignant tumor cells, CSCs are generated by acquiring tumorigenicity through abnormal genetic diversity, epigenetic modification, and signal transduction.^34^ In the present study, a HFD resulted in increased CSCs properties and Wnt activity in colonic crypts. These results may add understanding of the mechanisms by which ISCs can become tumor-initiating cells under a HFD. The CD44v isoforms are a critical marker for CRC, and their overexpression is a poor prognostic factor and predictor of metastasis in patients with CRC.^26^^,^^35^ Previous studies have demonstrated an association between CD44v expression and the Wnt signaling pathway in ISCs. For example, CD44v expression was identified in Lgr5^+^ ISCs and contributes to adenoma formation in *Apc*^*Min*/+^ mice as a Wnt signal target gene.^36^^,^^37^ Furthermore, CD44 supports the activation of Wnt signaling by regulating membrane localization of the Wnt receptor LRP6.^38^ These results imply that CD44 is related to Wnt signal activity, a risk factor for CRC. Because HFD induced hyperactivation of Wnt signaling in colonic crypts, CD44 overexpression may be an important indicator of intestinal tumorigenesis caused by HFD.

The stem cell niche has duplicity in that it regulates stem cell function and homeostasis while also driving to tumor progression and heterogeneity within the CSC niche.^39^ In particular, the formation and maintenance of CSCs have reportedly been regulated extrinsically by the CSC niche.^40^^,^^41^ We have demonstrated that overproduction of Wnt2b from HFD-derived MSCs augments the expression of Wnt target genes and CSCs markers in colonic crypts. Other data support these results, suggesting that exosomal Wnt ligands from CRC-derived CAFs activate canonical Wnt signaling in cancer cells and subsequently induce CSCs clonogenicity.^42^ Previous studies have also highlighted that carious CAF-derived factors such as hepatocyte growth factor and osteopontin can induce Wnt signal activity and promote CSCs clonogenicity.^26^^,^^41^ Furthermore, CD44v expression is triggered by factors secreted from CAFs, which subsequently increases the metastatic potential of CRC.^26^ Taken together, colonic MSCs may be involved in HFD-induced CRC by contributing to the transformation of ISCs to CSCs and mediating hyperactivation of Wnt signaling.

MSCs are activated into CAFs by signals such as growth factors and chemokines.^43^ In this study, RNA-seq data showed that HFD-derived MSCs had CAF-like properties with predominant CXCR4 and IL1RL1 expression. The autocrine CXCR4/CXCL12 pathway is known to activate MSCs into tumor-promoting CAFs^29^^,^^43^ and subsequently increases survival, migration, and cytokine secretion through PI3K/AKT signaling.^44^ Moreover, data have shown that MSCs adjacent to intestinal tumors express IL1RL1 and interleukin 33, which can induce activation of tumor-promoting MSCs.^30^ Transcription factor SOX2-expressing MSCs were presented in the CRC-associated CAFs population, which stimulated tumor progression.^31^ Therefore, we speculate that through diverse mechanisms, HFD activates MSCs into CAF-like cells.

Compared with normal MSCs, CAFs are heavily involved in proliferation and metabolism and increase the production of ECM components and adhesion molecules.^45^, ^46^, ^47^ Because of the interaction between the PI3K-Akt pathway and ECM components and cell adhesion molecules,^48^ the activation of this pathway in HFD-derived MSCs may induce the characterization of CAFs. Furthermore, metabolic changes in HFD-derived MSCs are consistent with a previous study that showed that PDGF- and TGF-β-induced CAFs undergo metabolic reprogramming.^49^ The aerobic glycolysis pathway is subsequently up-regulated, causing an increase in lactate production.^49^ Moreover, mitochondrial OXPHOS activity is higher in ISCs than Paneth cells, and OXPHOS demand for ISCs was satisfied by lactate produced by Paneth cells with high glycolytic activity.^50^ Therefore, increased glycolytic metabolite in HFD-derived MSCs may affect ISC function. In line with our results, HFD-induced obesity increases differentiation of adipose-derived MSCs into myofibroblasts and production of ECM components, which promote breast tumorigenesis.^15^ Although our current study focused on colonic MSC-induced Wnt2b overproduction, these CAF-like activities may also contribute to CRC incidence through a HFD. Our results suggest that targeting MSCs in the tumor microenvironment or CRC may act as a potential therapeutic strategy.

Gut microbiota are partially formed by and regulate the metabolism of BAs in symbiosis.^32^^,^^51^ CA feeding has reportedly expanded Firmicutes phylum, which subsequently increased the conversion of CA to secondary BAs (DCA) through the 7α-dehydroxylation reaction.^52^^,^^53^ In addition, previous research has shown that various *Bacteroides* strains perform deconjugation of BAs and 7-keto-DCA/7-dehydro-CDCA biosynthesis, which are important for secondary BA production.^54^ In particular, *Bacteroides vulgatus* deconjugates BAs through BSH activity.^32^ Thus, these studies may support the increase of deconjugated and secondary BAs under conditions of a HFD. Among the BAs metabolized by bacteria, CDCA and DCA were known as cytotoxic BAs that cause DNA damage and subsequently CRC.^55^, ^56^, ^57^ FXR, a BA-responsive receptor, has a high affinity for CDCA and DCA among the natural ligands.^32^ The regeneration of intestinal crypts through inhibition of prostaglandin E2 promoted by the activation of FXR signaling in colonic MSCs has also been reported.^57^ Our data demonstrate that CDCA and DCA induce Wnt2b production and CAF-like properties from colonic MSCs through activation of FXR signaling. Hence, BA modulation of ISC niche cellular function, such as MSCs, and abnormal function of colonic MSCs by BAs-FXR signaling may act as a risk factor for CRC.

In conclusion, our study demonstrates that aberrant MSCs were induced by a HFD drive hyperproliferation and transition of ISCs to CSCs in the colon. Furthermore, we suggest that BAs hold potential in regulating the function of colonic MSCs. These findings contribute to the improved understanding of the mechanisms by which a HFD may induce CRC and also offer another role for gut microbiota in regulating the function of the ISC niche.

## Materials and Methods

### Mice and Diet

C57BL/6N mice were purchased from Orient Bio (Seongnam, South Korea), and Lgr5-EGFP-IRES-CreERT2 mice and C57BL/6J-*Apc*^*Min*/+^ mice were purchased from Jackson Laboratory (Bar Harbor, ME). All mice were housed under specific pathogen-free conditions in the animal facility at Asan Medical Center, where they received water ad libitum. Three different types of diets were used throughout the experiment: standard normal chow (Purina 5057; Purina, St Louis, MO), PD (D10012G; Research Diets Inc, New Brunswick NJ), and 60% kcal HFD (D12492; Research Diets Inc). Mice received their respective food ad libitum from the age of 6 weeks until at least 2 months. Mice were sex- and age-matched between groups. *Apc*^*Min*/+^ mice with spontaneous intestinal adenoma after housing for 15 weeks were used for colon cancer organoids culture.

### Experimental Inflammation-Induced CRC Model

Mice were given a single intraperitoneal injection of AOM (10 mg/kg body weight; Sigma-Aldrich, St Louis, MO) combined with 3 cycles of dextran sulfate sodium treatment as illustrated in Figure 1*E*.

### Histology

Intestines were rolled using the Swiss roll technique and fixed in 4% paraformaldehyde. They were then dehydrated through a chain of graded ethanol baths and embedded in paraffin. The paraffin-embedded blocks were cut into 5-μm sections and stained with hematoxylin-eosin or periodic acid-Schiff. For the periodic acid-Schiff staining, tissue section slides were deparaffinized and rehydrated and placed in 1% periodic acid solution at room temperature (RT) for 5 minutes. Slides were then washed and immersed in Schiff’s reagent for 15 minutes and stained with hematoxylin.

### Histologic Scoring

Histologic grade was scored by blinded investigators through a scoring system as described previously^58^: the severity of inflammation (0, none; 1, slight; 2, moderate; 3, severe), the extent of injury (0, none; 1, mucosa; 2, mucosa and submucosa; 3, transmural and epithelium lost), and crypt damage (0, none; 1, basal one-third damaged; 2, basal two-thirds damaged; 3, only surface epithelium intact; 4, entire crypt and epithelium lost). Finally, the percentage of involvement by the disease conditions was quantified in these indicators (0, 0%–25%; 1, 26%–50%; 2, 51%–75%; 3, 76%–100%).

### Immunofluorescence Staining

Intestines were prepared by the Swiss roll technique for the frozen section and fixed in 4% paraformaldehyde. Tissues were then dehydrated stepwise with 15% and 30% sucrose in phosphate-buffered saline (PBS). Thereafter, rolled tissues were embedded in optimal cutting temperature compound (Sakura Finetek USA, Torrance, CA), frozen, and sliced to 5 μm, and tissue sections were fixed in −20°C acetone for 5 minutes. Paraffin or cryosections were permeabilized in PBS containing 0.5% Triton X-100 at RT for 20 minutes and blocked with 5% bovine serum albumin in PBS at RT for 1 hour. Tissue sections were then stained with primary antibodies overnight at 4°C. After washing with PBS, tissue sections were incubated with secondary antibodies at RT for 1 hour, stained with 4′, 6-diamidino-2-phenylindole (DAPI; Thermo Fisher Scientific, Waltham, MA) for 2 minutes at RT, and mounted with PermaFluor mountant (Thermo Fisher Scientific). To analyze the immunofluorescence of intestinal MSCs, plated MSCs were fixed with 4% paraformaldehyde in PBS. After MSCs were washed in PBS, cells were permeabilized in PBS containing 0.5% Triton X-100 at RT for 30 minutes. After staining, fluorescent images of all samples were captured on an LSM 710 confocal microscope (Carl Zeiss, Oberkochen, Germany). The following antibodies were used for staining: anti-Ki67 (BioLegend, San Diego, CA; clone 11F6), anti-CD44s-v (Abcam, Cambridge, UK; IM7) anti-β-catenin (BD Biosciences, Franklin Lakes, NJ; clone 14), anti-gp38 (R&D Systems, Minneapolis, MN), anti-CD90 (Abcam; IBL-6/23), and anti-Wnt2b (Abcam; clone EPR13386), Alexa fluor 546-conjugated donkey anti-rat IgG (Thermo Fisher Scientific), Alexa fluor 488-conjugated goat anti-mouse IgG (Abcam), Alexa fluor 488-conjugated donkey anti-rat IgG (Life Technologies, Carlsbad, CA), and Alexa fluor 647-conjugated donkey anti-goat IgG (Abcam).

### RNA in Situ Hybridization

According to the manufacturer’s instructions, the intestines were fixed in 4% paraformaldehyde, embedded in paraffin, and used for RNA in situ hybridization (RNAscope 2.5 HD Detection kit; Advanced Cell Diagnostics, Newark, CA). *Axin2*, *Wnt2b*, *Wnt2*, *Wnt3*, *Wnt4*, *Wnt5a*, *Wnt6*, and *Wnt9b* probes were purchased from Advanced Cell Diagnostics and hybridized to their target mRNA.

### Crypt Isolation

First, fat and adherent connective tissues were removed to obtain crypts from the colon. Colon tissues were then opened longitudinally, washed with cold PBS without magnesium chloride and calcium by vigorously shaking, and cut into approximately 1-cm sections. Tissue pieces were incubated in PBS containing 5 mmol/L EDTA/gentamicin (Gibco, Amarillo, TX) at 37°C for 30 minutes. Tissue pieces were washed with PBS, vigorously shaken, and then filtered by a 70-μm cell strainer (BD Falcon, Franklin Lakes, NJ) to isolate the intestinal crypts.

### Organoids Culture and Measurement

Colonic crypts were pelleted and resuspended with TrypLE Express (Thermo Fisher Scientific) for 15 minutes at 37°C to dissociate crypts as described previously.^52^ Dissociated colonic crypts were counted by a hemocytometer and embedded (2 × 10^4^ cells/well) in Matrigel (Corning, Corning, NY). Matrigel containing crypts were plated at 50 μL per well onto a flat-bottom 24-well plate (Thermo Fisher Scientific) and cultured in WENR medium containing B27 supplement (Thermo Fisher Scientific), N2 supplement (Thermo Fisher Scientific), N-acetyl cysteine (Sigma-Aldrich), EGF (Thermo Fisher Scientific), Noggin (R&D Systems), Wnt3a-conditioned medium, R-spondin-1-conditioned medium, Antibiotic-Antimycotic (Thermo Fisher Scientific), HEPES (Gibco), GlutaMAX (Gibco), and advanced Dulbecco modified Eagle medium [DMEM]/F12 (Thermo Fisher Scientific). Y-27632 (Sigma-Aldrich) and Jagged-1 (AnaSpec, Fremont, CA) were added to WENR medium for 3 days after the start of the culture, as described previously.^59^ To manipulate the cancer organoids, colonic crypts from *Apc*^*Min*/+^ mice were isolated and cultured in EN medium without Wnt3a and R-spondin-1. The culture medium was changed every 2–3 days. Organoids were mechanically disrupted by pipetting for subculture or further incubated in TrypLE Express Enzyme at 37°C for 15 minutes. Organoids were randomly photographed using an inverted microscope (Carl Zeiss), and each photo was analyzed using ImageJ software (NIH, Bethesda, MD) to measure the surface area. The perimeters for area measurements were defined manually by automated ImageJ software.

### Treatment of Fatty Acids and Cecal Microbial Products

The following fatty acids have been applied to both organoids and MSCs: palmitic acid (30 μmol/L; Sigma-Aldrich), oleic acid (30 μmol/L; Sigma-Aldrich), stearic acid (30 μmol/L; Sigma-Aldrich), or lipid mixture (1%; Sigma-Aldrich). All fatty acids were conjugated with bovine serum albumin. For CMP, cecal content was lyophilized, and the powder was dissolved in the advanced DMEM/F12 medium and vigorously vortexed for 1 hour at 4°C. After centrifugation, the supernatant was filtered using a 0.2-μm syringe filter (PALL) and treated into MSCs with titration.

### MSCs Culture

Colonic MSCs were isolated as described previously.^60^ In brief, the remaining colon tissues after crypt isolation were chopped and added with DMEM medium (Gibco) containing 10% heat-inactivated fetal bovine serum (Gibco), gentamycin/streptomycin, and collagenase type IV (Sigma-Aldrich), and incubated for 1 hour at 37°C with shaking (200 rpm). Dissociated tissues were shaken vigorously, and supernatants were passed through a 70-μm cell strainer (BD Falcon). Cell pellets were collected by centrifuge at 280*g* for 10 minutes, washed, and cultured in DMEM containing 10% fetal bovine serum (Gibco), 2 mmol/L GlutaMax (Gibco), and 1% Antibiotic-Antimycotic (Thermo Fisher Scientific). MSCs were subcultured to obtain high purity cells.

### Flow Cytometry

Isolated MSCs were blocked with solution containing 0.2% bovine serum albumin in PBS and stained with Live/Dead cell stain kit (Thermo Fisher Scientific) and antibodies: anti-CD45 (BD Biosciences; clone 30-F11), anti-CD31 (BD Biosciences; clone MEC13.3), anti-EPCAM (eBioscience, Hatfield, UK; clone G8.8), anti-gp38 (BioLegend; clone 8.1.1), and anti-CD90 (eBioscience; clone 53-2.1). Flow cytometry was performed using a FACS CANTO II (BD Biosciences), and files were analyzed using FlowJo software (Tree Star, Ashland, OR).

### Co-culture With Organoids and MSCs

MSCs (2 × 10^4^/well) were suspended in Matrigel matrix in the presence of organoids (2 × 10^4^/well) obtained from NCD-fed control mice or *Apc*^*Min*^/J mice. After the Matrigel polymerization, cells were cultured in ENR medium (without Wnt3a) or EN medium. C59 (10 μmol/L; Abcam) was treated in ENR medium at 7 days after culture to inhibit Wnt secretion.

### Real-Time Quantitative Polymerase Chain Reaction

Total RNA was extracted using the RNeasy mini kit (Qiagen, Hilden, Germany). RNA was converted to cDNA with Superscript II reverse transcriptase and oligo (dT) primer (Thermo Fisher Scientific). cDNA was used as the template for real-time quantitative polymerase chain reaction (qPCR) performed using SYBR green chemistry (Affymetrix, Santa Clara, CA) on an ABI 7500 Real-Time qPCR System (Applied Biosystems, Waltham, MA). The primer sequences used for real-time qPCR are as follows: *Lgr5*: forward 5´-CCTGTCCAGGCTTTCAGAAG-3´, reverse 5´-CTGTGGAGTCCATCAAAGCA-3´; *CD44*: forward 5´-TCCTTCTTTATCCGGAGCAC-3´, reverse 5´-ACGTCTCCTGCTGGGTAGC- 3´; *Reg4*: forward 5´-CTGGAATCCCAGGACAAAGAGTG-3´, reverse 5´-CTGGAGGCCTCCTC AATGTTTGC-3´; *c-Kit*: forward 5´-TCATCGAGTGTGATGGGAAA-3´, reverse 5´-GGTGACT TGTTTCAGGCACA-3´; *Egf*: forward 5´-TTCTCACAAGGAAAGAGCATCTC-3´, reverse 5´-GTCCTGTCCCGTTAAGGAAAAC-3´; *Egfr*: forward 5´-GCTGGTGTTGCTGACCGCG-3´, reverse 5´-GGGTGAGCCTGTTACTTGTGCC-3´; *ErbB2*, forward 5´-GCAAGCACTGTCTGCCA TGC-3´, reverse 5´-GGGCACAAGCCTCACACTGG-3´; *Notch1*: forward 5´-GCTGCCTCTTTGA TGGCTTC-3´, reverse 5´-CACATTCGGCACTGTTACAG-3´; *Dll1*: forward 5´-CAGGACCTTC TTTCGCGTAT-3´, reverse 5´-AAGGGGAATCGGATGGGGTT-3´; *Dll4*: forward 5´-TTCCAGG CAACCTTCTCCGA-3´, reverse 5´-ACTGCCGCTATTCTTGTCCC-3´; *Hes1*, forward 5´-CCAGC CAGTGTCAACACGA-3´, reverse 5´-AATGCCGGGAGCTACTTTC-3´; *Fzd5*: forward 5´-CCGC ATACCACAAGCAAG-3´, reverse 5´-GCATCAGCACCAAGAAGG-3´; *Ctnnb1*: forward 5´-AT GGAGCCGGACAGAAAAGC-3´, reverse 5´-TGGGAGGTGTCAACATCTTC-3´; *Axin2*, forward 5´-AACCTATGCCCGTTTCCTCT-3´, reverse 5´-GAGTGTAAAGACTTGG TCCA-3´; *Gsk3β*: forward 5´-ACCGACAACCACCTCCTTTG-3´, reverse 5´-TCACAGGGAGTGTCTGCTT G-3´; *Cyclin D1*: forward 5´-GCGTACCCTGACACCAATCTC- 3´, reverse 5´-CTCCTCTTCGCA CTTCTGCTC-3´; *c-Myc*: forward 5´-TGAGCCCCTAGTGCTGCAT-3´, reverse 5´-AGCCCGAC TCCGACCTCTT-3´; *Lef*: forward 5´-TGTTTATCCCATCACGGGTGG-3´, reverse 5´-CATGGA AGTGTCGCCTGACAG-3´; *Apc*: forward 5´-AGCCATGCCAACAAAGTCATCACG-3´, reverse 5´-TTCCTTGCCACAGGTGGAGGTA-3´; *Epcam*: forward 5´-ATGGACCTGAGAGTGAAC GG-3´, reverse 5´-CACGGCTAGGCATTAAGCTC-3´; *CD24*: forward 5´-GTTGCACCGTTTCCC GGTAA-3´, reverse 5´-CCCCTCTGGTGGTAGCGTTA-3´; *CD166*: forward 5´-ATGGCATC TAAGGTGTCCCCT-3´, reverse 5´-CTGAGTTGACAGTGTACCATCC-3´; *CD29*: forward 5´- ATGCCAAATCTTGCGGAGAAT-3´, reverse 5´-TTTGCTGCGATTGGTGACATT-3´; *Aldh1a1*: forward 5´-ATACTTGTCGGATTTAGGAGGCT-3´, reverse 5´-GGGCCTATCTTCC AAATGAACA-3´; *Ephb2*: forward 5´-TCATCGCTGTGGTCGTCATTG-3´, reverse 5´-GTCC GCTGGTGTAGTGTTGTAG-3´; *Wnt2b*: forward 5´-CCGTGTAGACACGTCCTGGT-3´, reverse 5´-TGATGTCTGGGTAGCGTTGA-3´.

### RNA-seq Analysis

Total RNA was extracted using the RNeasy mini kit (Qiagen). A library was prepared with 1 μg of total RNA for each sample using the TruSeq Stranded mRNA LT Sample Prep kit (Illumina, San Diego, CA). The protocol was performed with polyA-selected RNA extraction, RNA fragmentation, random hexamer-primed reverse transcription, and 101 nt paired-end sequencing by the NovaSeq6000 platform (Illumina). The libraries were quantified using qPCR according to the qPCR Quantification Protocol Guide (KAPA Library Quantification Kit for Illumina Sequencing platforms) and qualified using the 2100 Bioanalyzer (Agilent Technologies, Santa Clara, CA). RNA-seq experiments and statistical analyses were performed by Macrogen Inc (Seoul, South Korea). The reads were pre-processed to remove low-quality and adapter sequences before analysis, and processed reads were aligned to *Mus musculus (mm10)* using HISAT v2.0.5. After alignment, raw data were obtained through the StringTie v1.3.3b to assemble aligned reads into transcripts and estimate their abundance. Filtered data were log_2_-transformed and subjected to quantile normalization. For pathway analysis, significant gene lists were performed on the basis of the KEGG pathway. For GSEA, the raw gene matrices count was normalized and performed with GSEA software using a pre-ranked dataset. *P* values in the GSEA data were estimated by 1000 permutations.

### 16s rRNA Sequencing for Metagenomic Analysis

Bacterial DNA was extracted from feces using QIAamp DNA stool mini kits (Qiagen). PCR amplification was performed using primers targeting the segment from the V3 to V4 regions of the 16S rRNA gene with extracted DNA. For bacterial amplifications, we used barcoded primers of 341F (5′-CCTACGGGNBGCASCAG-3′) and 805R (5′-GACTACNVGGGTATCTAATCC-3′). The amplifications were performed under the following conditions: initial denaturation at 95°C for 5 minutes, followed by 30 cycles of denaturation at 95°C for 30 seconds, primer annealing at 55°C for 30 seconds, and extension at 72°C for 30 seconds, with final elongation at 72°C for 5 minutes. The QIAquick PCR purification kit (Qiagen) was used to purify the purification of the amplified products. Equal concentrations of purified products were pooled, and short fragments (non-target products) were removed with an AMPure bead kit (Agencourt, Beverly, MA). The quality and product size were assessed on a Bioanalyzer 2100 (Agilent) using a DNA 7500 chip. Mixed amplicon sequencing was conducted by emulsion PCR and then deposited on picotiter plates. The sequencing was carried out at Chunlab (Seoul, South Korea) on a GS Junior Sequencing System (Roche, Basel, Switzerland) per the manufacturer’s instructions. Taxonomic cladogram was analyzed using LEfSe with a threshold 2 on the logarithmic LDA score.^61^

### Measurement of OCR and ECAR by XF24 Flux Analyzer

Primary cultured colonic MSCs were seeded at 4 × 10^4^ cells per well in XF24 cell culture microplates (Agilent Technologies) and cultured in MSC culture medium overnight. One hour before measurement, culture medium was replaced with OCR assay medium (minimal DMEM [Sigma-Aldrich] and supplemented with 20 mmol/L glucose [Junsei Chemical, Tokyo, Japan], 2 mmol/L GlutaMax [Gibco], and 5 mmol/L pyruvate [Sigma-Aldrich]) or ECAR assay medium (minimal DMEM and supplemented with 2 mmol/L GlutaMax) in the 37°C non-CO_2_ incubator. The Seahorse Bioscience XF24 analyzer (Agilent Technologies) was used to measure OCR and ECAR. For OCR measurements, 1 μmol/L oligomycin (Sigma-Aldrich), 1 μmol/L FCCP (Sigma-Aldrich), and 1 μmol/L rotenone and antimycin (Sigma-Aldrich) were injected.. For ECAR measurements, 10 mmol/L glucose, 2 μmol/L oligomycin, and 50 mmol/L 2-deoxy-D-glucose (Sigma-Aldrich) were injected. The parameters of glycolytic function, glycolytic capacity, glycolytic reserve, and non-glycolytic acidification were calculated using Seahorse Wave software V2.6.

### Analysis of FFAs Using Gas Chromatography–Tandem Mass Spectrometry

The cecal content was lyophilized by Freeze Dryer (Martin Christ, Munich, Germany) after pre-freezing overnight at –80°C. The powder was homogenized with cold methanol (MeOH) and internal standard solution (0.1 mg/mL myristic acid-d_27_). Sample solutions were acidified with HCl to 25 mmol/L final concentration and centrifuged. Supernatants were collected into fresh tubes, and iso-octane was added. The upper phase was collected after the liquid–liquid extraction process and dried under vacuum. The dried sample was combined with BCl_3_-MeOH, 12% (w/w, Sigma-Aldrich) at 60°C for 30 minutes. H_2_O and hexane were added sequentially, and the sample was mixed vigorously. The upper phase was collected after resting the sample for 5 minutes. Anhydrous sodium sulfate was added, and the supernatant was then ready for gas chromatography–mass spectrometry analysis. Fatty acid methyl esters (Sigma-Aldrich) were used to generate calibration curves without derivatization and analyzed with the GC-MS system (Agilent 7890A/5975C) using capillary columns (HP-5MS, 30 m × 0.25 mm × 0.2 μm). Electron impact ionization was used in positive ion mode. One μL of injection volume and split mode (ratio 10:1) were used. Extracted ion chromatogram by the specific fragment ion per fatty acid was used to quantify the negative ion mode. Data analysis was performed using MSD ChemStation software (Agilent E02.02.1431).

### Analysis of BAs Using Liquid Chromatography-Tandem Mass Spectrometry

Feces were collected through individual housing for 1 day and lyophilized by Freeze Dryer (Martin Christ) after pre-freezing overnight at –80°C. Ten mg of freeze-dried feces was homogenized with 50% MeOH. An internal standard solution (50 μL of 1 μmol/L cholic acid-d_5_ solution) and 5% NH_4_OH in cold acetonitrile (ACN) were added to the homogenate. The solution was then incubated for 1 hour at RT with gentle shaking. Fifty μL of serum was mixed with cold ACN and an internal standard solution. The sample solution was centrifuged at 13,000 rpm for 10 minutes at 4°C. The supernatant was collected and dried using a vacuum centrifuge. The dried matter was stored at –20°C and reconstituted with 50% MeOH before liquid chromatography-tandem mass spectrometry analysis. Lipid levels of BAs were determined using a liquid chromatography-tandem mass spectrometry system equipped with a 1290 HPLC (Agilent) and QTRAP 5500 (AB Sciex, Toronto, Canada). A reverse-phase column (Pursuit5 C18, 150 × 2.1 mm) was used with mobile phase A (7.5 mmol/L ammonium acetate, pH4 using 10 mol/L acetic acid) and mobile phase B (5% CAN in MeOH). The liquid chromatography was run at 200 μL/min and 24°C. Multiple reaction monitoring was performed under negative ion mode, and the extracted ion chromatogram corresponding to the specific transition for each BA was used for quantification. The calibration range for each lipid was 0.1–10,000 nmol/L (r^2^ ≥ 0.99). Data analysis was performed using either Analyst 1.5.2.

### Total Bile Acid Analysis

According to the manufacturer’s instructions, total BA was measured in serum and feces using the total BA assay kit (Crystal Chem Inc, Elk Grove Village, IL). Fifty mg of the lyophilized feces was extracted in 4 mL of 75% (v/v) ethanol at 50°C with gentle agitation for 2 hours. Fecal extracts after centrifugation were used to quantify the total BAs.

### BSH Activity Assay

After feces were ground with PBS in a 100-μm strainer, the filtered solution was dropped onto DISK mounted on MRS agar containing taurodeoxycholic acid (Sigma-Aldrich). The activity of BSH was analyzed by measuring the diameter of the precipitated colonies.

### Statistical Analysis

GraphPad Prism software (GraphPad, San Diego, CA) was used for statistical analyses. Significant differences between the 2 groups were analyzed with 2-tailed unpaired *t* test. Multiple groups were analyzed by one- or two-way analysis of variance (ANOVA), followed by Bonferroni post hoc test (∗*P* < .05, ∗∗*P* < .01, ∗∗∗*P* < .001).
